# Lessons and Future Directions for a Gender Equity Pledge Campaign

**DOI:** 10.1089/whr.2022.0111

**Published:** 2023-05-29

**Authors:** Elizabeth H. Ellinas, Leon J. Gilman, Amy H. Farkas

**Affiliations:** ^1^ Center for the Advancement of Women in Science and Medicine, Department of Anesthesiology, Medical College of Wisconsin, Milwaukee, Wisconsin, USA.; ^2^ Office of Diversity and Inclusion, Center for the Advancement of Women in Science and Medicine, Medical College of Wisconsin, Milwaukee, Wisconsin, USA.; ^3^ Division of General Internal Medicine, Medical College of Wisconsin, Wauwatosa, Wisconsin, USA.

**Keywords:** culture, equity, women, faculty, learners, staff

## Abstract

**Background:**

In November 2019, the IWill gender equity pledge campaign called individuals in a health sciences university to make public pledges for gender equity and fostered meaningful dialog to alter mental models and power dynamics. Over 1400 staff, faculty, and students chose 1 of 18 pledges or created their own.

**Methods:**

A follow-up, mixed-methods survey was sent to 1405 participants in July 2020.

**Results:**

Fifty-six percent (*n* = 769) responded. Over 70% endorsed fulfilling their pledge and believed they had the power to promote equity. Men were significantly more likely to endorse fulfilling their pledge, and men and learners endorsed having the power to create change at significantly higher rates than women. Key barriers included time, support for completion, and nonconducive culture or hierarchy. Key supports included personal reminders, self-reflection, and support from a partner, community, or leader. Top reasons for participation in the campaign included fairness or justice, being part of a community, team diversity as an inherent good, and the sense that the Medical College of Wisconsin's (MCW) should be a leader in gender equity.

**Conclusion:**

The IWill campaign successfully encouraged faculty, staff, and learners to reflect upon and engage in equity work. Key learnings included the need to streamline administrative support while building a sense of community around equity, and the further work needed to engage leaders and directly support not just individual but also departmental and institutional efforts in gender equity.

## Introduction

Despite decades of near-equal numbers in medical-school matriculation^1^ and assistant professorships,^2^ women in academic medicine continue to lag behind men in upper level positions.^3^ Women face barriers ranging from a lack of mentorship and sponsorship^4^ to workplace microaggressions and macroaggressions^5^ and home life inequities,^6,7^ making culture and climate frequent areas of research and concern. A variety of modalities—from educational workshops and campaigns to structured departmental supports—have been employed to promote an equitable culture with various levels of success.^8–11^

In 2019, the Medical College of Wisconsin's (MCW) Center for the Advancement of Women in Science and Medicine (AWSM) began its culture initiatives with the IWill MCW gender equity pledge campaign.^12^ The campaign was based on the framework of change developed by Kania, et al. which describes three change levels and six interdependent conditions that are must be addressed to effect system change: explicit (policies, practices, and resource flows), semi-implicit (relationships and power dynamics) and implicit (mental models).^13^ The IWill campaign was focused on the semi-implicit and implicit levels, seeking to shift power dynamics and build relationships between stakeholders, while also asking people to examine their mental models and habits to transform deeply held (and sometimes invisible) beliefs toward equitable practices.

The IWill campaign asked individuals to make personal, call-to-action public pledges for gender equity, with the goal to foster an environment in which all genders can thrive.^12^ Participants could choose from 18 pledges (or create their own pledge) in five pledge categories: becoming a workplace ally, mitigating unconscious bias, creating belonging, supporting parents, and creating a sexual and gender harassment-free environment. For example, one pledge in the supporting parents pledge category was “IWill be aware of maternal bias and work to diminish it.” IWill hoped to engage 1350 participants who could be innovators and early adopters of ideas—believing that these individuals would be the catalyst for change.^14^ The IWill campaign met that goal: over 1400 staff, faculty, and students pledged in the campaign.

The IWill team knew that the campaign risked a lip-service approach to pledging, and drew on pledge literature to increase campaign engagement and pledge fulfillment, including making pledges public,^15,16^ supporting pledges with information and action plans,^17^ and having a variety of pledges to choose from.^18^ Beyond the effectiveness of our fulfillment strategies, the IWill team sought pledge makers' opinions regarding barriers and supports to successful pledge completion, their feelings regarding success of the campaign overall, and their own empowerment to achieve gender equity.

## Methods

This study was approved by the Medical College of Wisconsin Institutional Review Board.

In the initial IWill campaign, a total of 1405 people made pledges between the fall of 2019 and winter 2020, representing 16% of the eligible population. A follow-up survey was designed to help the IWill team reflect on the pledge process, the pledges themselves, participants' motivations for participating in IWill, and the impact of IWill on the campus environment. Quantitative items consisted of five-item Likert scales with answer choices generally ranging from (1) Strongly Disagree to (5) Strongly Agree. Qualitative items were short answer open text responses.

Responses to an open-text “why participate” question in the initial pledge campaign were analyzed and a list was created, which appeared as a rank-list question in the follow-up survey. A full text of the survey appears in Supplementary Appendix SA. The survey was sent to all pledge makers through an individual email link using the REDCap platform.^19^ The survey was open from May 19 to June 30, 2020, with reminder emails sent weekly. Like the initial pledge-collection instrument, this survey was confidential, but not anonymous, allowing demographics to be linked from the original pledge survey to the follow-up survey.

Respondents were divided into faculty, learners, and staff. “Staff” included any employee of the health sciences university, who was not a faculty or learner. Faculty and staff roles were further divided into men and women. Learners were not divided due to small group size. Respondents identifying as neither women nor men were removed from quantitative analysis due to small group size (less than 5), but were included in qualitative analysis. The IWill team acknowledges that at times, in this article, we refer to only two gender groups (men and women), those binary categories do not fully encompass how many individuals express their gender identity.

Responses were analyzed using both quantitative and qualitative methods. Quantitative responses were evaluated using SPSS 28 and included Bonferroni's correction for multiple comparisons. Qualitative results were analyzed using Consensual Qualitative Analysis (CQA).^20^ On the CQA team, EHE and LJG served as primary members to consider core ideas, themes, and cross analysis; AHF served as CQA auditor.

## Results

Of the 1405 people who made initial pledges, 769 (56%) completed the follow-up survey. Survey participation (50% women faculty and 79% women staff) resembled the MCW population (42% women faculty and 76% women staff overall), and included 160 men faculty (21%), 160 women faculty (21%), 291 women staff (38%), 76 men staff (10%), and 82 learners/trainees (11%).

### Pledge fulfillment

Six hundred sixty-three survey respondents (84%) strongly agreed or agreed they remembered their pledge, 643 (82%) reflected about equity, and 509 (65%) learned something new. More than 80% (628) of respondents reported “maybe” or “definitely” fulfilling their IWill pledge. People who agreed to share both their pledge and name publicly were significantly more likely to endorse fulfilling their pledge than people who shared just their name or pledge alone or kept their pledge and name private [*χ*^2^(1, 775) = 24.702, *p* < 0.001]. Pledge category showed no statistically significant relationship to pledge fulfillment [*F*(5, 758) = 2.001, *p* = 0.076]. Men were significantly more likely to endorse fulfilling their pledge than women *t*(773) = 4.073, *p* < 0.001. More specifically, one-way analysis of variance (ANOVA) using gender-role groups indicated that this was largely due to differences between men faculty and women staff (*p* < 0.001) (Table 1).

**Table 1.** Means and Analysis of Variance Results by Gender-Role Group

Question	Means						ANOVA results	
	Women faculty (*n* = 160)	Men faculty (*n* = 160)	Women staff (*n* = 291)	Men staff (*n* = 76)	Learner/trainee (*n* = 82)	All (*n* = 769)	F (df)	*p*
I learned something new about gender equity.	3.56	3.77	3.69	3.72	3.79	3.69	*F*(4, 762) = 1.62	0.167
I was able to take some time to reflect about gender equity in my environment.	3.92	4.13	4.00	4.11	4.20	4.04	*F*(4, 761) = 2.64	0.033
I acted to promote gender equity.	3.83	4.01	3.89	4.00	4.00	3.93	*F*(4, 756) = 1.56	0.184
I remembered my IWill pledge.	4.12	4.20	4.05	4.21	3.94	4.10	*F*(4, 763) = 1.85	0.117
I have become aware of a gender inequity that I did not see before.	3.26	3.49	3.46	3.51	3.54	3.44	*F*(4, 757) = 1.68	0.152
I was able to fulfill my IWill Pledge	4.13	4.34^a^	3.92^a^	4.26	4.18	4.11	*F*(4, 759) = 5.70	**<0.001**
I have the power to create gender equity in my work environment.	3.79^b^	4.21^b^	3.76^b^	4.16^b^	4.09^b^	3.93	*F*(4, 737) = 9.31	**<0.001**
I was able to gain what I hoped I would from IWill MCW.	3.43	3.64	3.44	3.69	3.66	3.53	*F*(4, 748) = 3.60	0.006
I believe that IWill MCW has improved gender equity at MCW.	3.33^c^	3.55	3.38	3.66^c^	3.59	3.45	*F*(4, 751) = 4.06	**0.003**
I would like for there to be another opportunity to make a gender equity pledge.	3.65	3.59	3.71	3.69	3.99	3.70	*F*(4, 744) = 2.58	0.036
If there is another opportunity to pledge, the pledges should be the same as the previous set.	3.16	3.28	3.12	3.12	3.17	3.17	*F*(4, 736) = 1.13	0.342
Did you encourage anyone to make a pledge? (Yes/No)	0.36	0.39	0.48	0.41	0.53	0.43	*F*(4, 734) = 2.56	0.037

Bolded items indicate significance in the omnibus test with *p* < 0.004 considered significant after applying the Bonferroni correction for 12 total ANOVA tests; Letters indicate significance in *post hoc* tests.

^a^ Women staff significantly different than men faculty (*p* < 0.001).

^b^ Women faculty significantly different than men faculty (*p* < 0.001) and men staff (*p* = 0.003). Women staff significantly different than learners/trainees (*p* = 0.033), men faculty (*p* < 0.001), and men staff (*p* = 0.005).

^c^ Women faculty significantly different than men staff (*p* = 0.029).

ANOVA, analysis of variance; MCW, Medical College of Wisconsin's.

Respondents shared both how they fulfilled their pledges, and barriers and supports to pledge fulfillment. Pledge-fulfillment methods fit themes related to Kania's Systems of Change framework,^13^ including explicit (structural) changes, semi-implicit (relational) changes, and implicit (individual mental model) changes. The IWill campaign was aimed at relational and mental-model changes, and the ways people fulfilled their pledges typically fell into one of those two themes. For example, one pledge maker described “stepping aside” on a project to help a woman succeed, sharing “I know it will be a golden opportunity for several female colleagues to shine.” (man, staff). Others discussed awareness as a way of changing their mental models, “I am listening to their stories and learning the issues that need to be resolved in my department” (man, faculty). Additional quotes regarding pledge fulfillment are available in Table 2.

**Table 2.** Additional Quotes Regarding Ways People Fulfilled Their Pledges

Ways pledges fulfilled		
Gender	Role	Quote
Explicit: Policies, best practices, or procedures. Directing resources toward goals.		
Man	Staff	(I) noticed that there was a deficiency in equality, and a violation of the ACA [regarding women expressing breast milk]. (I) brought my concerns to [the Ombuds Office] and worked with them to understand the requirements of the ACA and to identify [potential lactation-room] locations on campus.
Man	Faculty	Specific discussions occurred and more attention was paid to what appeared to be gender compensation inequities.
Woman	Staff	In my department most of our senior leaders are male. I brought this to the attention to my senior leadership group and we incorporated a process to obtain needed feedback from our female leaders to create balance in leadership guidance.
Semiexplicit: Power dynamics and increasing connections and improving relationships		
Man	Faculty	I declined an invitation to be a subject matter expert on an all-male panel… and suggested a more-qualified female colleague to be on the panel in my place, an invitation she accepted.
Woman	Staff	Selecting a pledge that encouraged [me to] bring others into discussions and decisions was a good task for me. A nice way to stretch.
Woman	Staff	I [am] speaking more candidly and openly with leaders and peers when asked for input… and striving to acknowledge my own accomplishments by not shying away from bringing them up in the appropriate context and by recognizing their full impact.
Implicit: Changing mental models—how I think.		
Man	Faculty	Increased amount of empathy considering the work situation of mothers around me, this has become a real world exercise as my wife and I alternate the anchor parent role.
Woman	Faculty	Reading through other pledges increased my awareness in general of issues I may not have otherwise put into words.
Woman	Staff	Gender equity issues demand that we ask more questions about a situation rather than just adding up hours worked or absences logged. It drove me to ask questions of myself like ‘How could this situation bring about positive change for my team? What is the story behind the numbers?… Lesson learned? Ask why. Ask how.

ACA, Affordable Care Act.

Respondents shared barriers to pledge fulfillment, including competing priorities, insufficient support for completion (e.g., reminders or activities), not having applicable situations to act on pledges, and feeling blocked by culture or hierarchy. The coronavirus disease 2019 (COVID-19) pandemic was a particularly salient distraction, and one respondent simply expressed that there were “too many excuses, all valid in their own way.” (man, staff) Others couldn't remember their pledge, or “lost focus on the pledge” (woman, staff) when working remotely. Related to focus, while more than 75% of respondents felt the informational materials (sent automatically by email after pledging) were useful, many suggested that more support from the IWill team was needed, sharing that “it would be helpful to have some reminders or accountability measures in place to check in after a few weeks or months” (woman, learner).

In the campaign, pledge makers were provided with a wide variety of pledges, including attending one event, starting book clubs, and engaging diverse teams. Some follow-up survey respondents reflected that their pledge was, by its nature, easy or difficult to fulfill, but not in ways the IWill team initially predicted. For example, some people who pledged to attend an event were stymied by COVID-19's event cancellations. Others, frequently women who pledged to work on their own behaviors (e.g., apologizing less or speaking up for themselves), felt like the pledge was “a lifelong commitment” further sharing that “I cannot say that I will ever completely fulfill my pledge” (woman, faculty).

People's experiences with systems or culture were highly varied, some suggesting that it was not a barrier in their department: “I already work in an environment that fully endorses, and indeed does not accept, gender inequity” (man, staff) or that systems' problems were a deeply engrained problem.

Several staff members commented that the power differential between staff and faculty was entrenched and not conducive to equity: “I am a staff member, not an MD and staff are viewed as lesser than” (woman, staff), or “I can support it, but I do not know that I can create it. Culture is generally (though not always) top down” (gender nonconforming, staff). Others viewed the situation through the lens of repercussions, saying that “[it is] very difficult for me to act on certain things because of the existing culture. I have no protection against political revenge” (woman, staff). Gendered expectations were also a concern: “our environment is such that women are expected to be kind, gentle, accommodating, and helpful. Speaking up feels it's in opposition to that. It's so normative” (woman, faculty).

Supports included pledge makers posting personal reminders and receiving support from others: “having a close personal friend hold me accountable and having witnessed persons struggle in the area of my pledge were key motivators in making my pledge personal” (man, faculty). In addition, several mentioned altering their mental models as a key to success: “self-reflection on my own career and leadership path and understanding that speaking up is important even if some have other viewpoints” (woman, staff). Additional quotes for barriers and supports are available in Table 3.

**Table 3.** Additional Quotes Regarding Barriers and Supports to Pledge Fulfillment

Barriers to pledge completion		
Gender	Role	Quote
No time or competing priority		
Woman	Faculty	I can't remember what it was. There are too many asks between patient load, research, emerge, etc. I have pledge fatigue.
Woman	Learner	The emotional turmoil, increased (unpaid) domestic responsibilities, and general disenfranchisement made it difficult to access my progress on the goal.
Woman	Faculty	The change in environment (virtual) eliminated the ‘normal’ environment. Had to learn virtual etiquette first before exploring how to include pledge goals.
Insufficient support		
Man	Learner	I felt like it was hard to be committed to this pledge when I was really only involved in it through email communication every so often.
Woman	Faculty	Maybe I missed them, but an opportunity to chat with others who made the same pledge would have been fun.
Woman	Learner	It would be helpful to have some reminders or accountability measures in place to check in after a few weeks or months
Woman	Staff	If you are hoping for participation in the community, you will have to bring these opportunities to us.
No applicable situations to act		
Woman	Staff	One aspect of my pledge was to be a role model for other women in terms of speaking up and valuing my voice. It's hard to gauge impact of this kind of ‘remote’ mentoring.
Woman	Faculty	It was not something that I came across frequently in my role. If there is rampant gender inequity on campus, I did not recognize it.
Man	Faculty	I think I have always been sensitive to this issue so this program does not enhance what I already believe and promote.
Culture or hierarchy		
Woman	Faculty	There is a strong hierarchy at MCW that when threatened, resists and retreats to deeply conditioned ways of responding.
Woman	Faculty	MCW is still male dominated and not all male leadership recognize when they are being sexist.
Woman	Faculty	I have tried and it is not looked upon well. As a woman leader you are ‘too strong’ ‘need to soften your approach’ and ‘people should look at you as a mothering figure.’
Pledge nature		
Woman	Staff	I don't know if I would say I ‘fulfilled’ my pledge only because I think these pledges have to be ongoing for the duration of our lives. I made progress on it, yes. There is so much to unpack and learn when it comes to gender equity and I believe it has to be an ongoing commitment.
Woman	Staff	The tasks required to complete my pledge were quite easy to accomplish.

Supports for pledge completion		
Gender	Role	Quote
Personal reminders		
Man	Faculty	Posted weekly reminder in my Outlook calendar ‘to do’ list of my pledge to work on daily
Woman	Faculty	Relating to the pledges of others was fun and helped hold me accountable. I also found the tip to print the pledge and put in on my computer monitor to be a helpful daily reminder
Man	Faculty	I wear my IWill MCW badge daily with pride and it also serves as a physical reminder.
Self reflections		
Woman	Faculty	When and how did I contribute to the problem? When and how could I have done more to address or prevent the problem?
Man	Faculty	Attended a focus group meeting, talked with my Department leader, talked with HR, and took parental leave myself.
Man	Faculty	I googled and read some other materials which confirmed what I know.
Support from a partner, community or leader		
Woman	Staff	Seeing other women faculty members display their pledges and buttons have my a sense of strong sisterhood despite being so low in the hierarchy at MCW. Around them, I felt I could open up with my thoughts on gender equity and they would be a willing participant in conversation.
Woman	Learner	Pledging with a partner or within a group was helpful for accountability and feeling supported in the process.

### Empowering people to create change

Five hundred seventy-six (74%) respondents endorsed promoting gender equity, while 575 (76%) believed they had the power to promote equity in their work environment. ANOVA *post hoc* comparisons among the five gender-role groups revealed women faculty endorsed having power to create gender equity at significantly lower rates than men faculty (*p* < 0.001) and men staff (*p* = 0.003). Women staff were also significantly less likely to feel empowered to create equity than learners (*p* = 0.033), men faculty (*p* < 0.001), and men staff (*p* = 0.005) (Table 1).

Qualitatively, while respondents cited culture or leadership as a hindrance to their power to create equity, others recounted their efforts to lead by example or shared their sense that they could be the tiny beginning to big change.

Some expressed frustration with framing the question in terms of power; a man faculty member wrote “This is poor word choice. Authority is one definition of power. Few have that here-I do not. I do have the power to set an example.” While a man learner countered, “We are all agents of change. You have power, and if your institution is setting the tone for change, you should be the change alongside them.” A woman learner added, “I believe that any small act in support of gender equity can make a change. And many small acts together can make an even greater change. No one person is too small to make a difference.” Examples of how this ripple effect could work were also shared:

“I made a conscious effort to address my female colleagues as ‘Dr.’ when we were not in casual or 1:1 settings, regardless of my relationship with them. When they would tell me that it was okay to use their first name, I would explain this pledge to them. On more than one occasion, a faculty member that I had this interaction with would reach out to me later and tell me that they had done some research and had no idea that this was an issue.” (woman, faculty)

Some felt that no matter how they acted, their acts alone would not be powerful enough: “I can do some things, e.g., no 7 am meetings, ensure pay equity, create opportunities, and support networking. These things, and others, help support gender equity but by themselves cannot ‘create’ equity and overcome more powerful external forces” (man, faculty).

Leaders were seen as the lynchpins of change, whose behaviors were critical to participants' success (or failure) in completing their pledge. For example, “my leadership supports [my pledge], which empowers me” (woman, staff), versus “my boss's micro and macro aggressions were too numerous, and the power differential was significant” (man, faculty). Leaders recognized that they have influence: “As a leader I can play an important role in gender equity” (man, staff). “I am strongly supported by leaders above me, and… I am well-positioned to affect gender equity… both to upper leadership as well as with my colleagues” (woman, faculty). Other leaders were less optimistic: “I have tried through my career to affect gender equity, and I do not see that much has changed. It is our [executive] leaders that have the real power. I mostly seem to be counseling others not to give up” (woman, faculty).

This lack of power was sometimes expressed as leader accountability: “I hope to see more equality from leaders who don't DO what they SAY. Many of our male leaders talk a good talk. They need to have their perspectives broadened” (woman, staff). A woman faculty surmised, “The challenge is that, often unconsciously, we work to maintain the status quo when it's working well for us. When men… are doing well, serving in leadership positions, moving along the ladder quite easily, they are unlikely to push for significant changes that could mean less power, privilege, and pay for themselves or their (male) friends.” One person summed up,” “My point is it will take multiple people doing this work on multiple levels” (woman, learner).

### Improving gender equity at MCW

Overall, participants were less enthusiastic the campaign improved gender equity with 352 (45%) agreeing or strongly agreeing IWill accomplished this goal. One hundred forty-eight men (55%) and 204 women (40%) agreed or strongly agreed there was improvement in gender equity, and 159 men (60%) and 244 women (49%) agreed or strongly agreed they got what they hoped for from the campaign. ANOVA results by gender role indicated a significant difference between men staff and women faculty regarding equity improvement secondary to the campaign (*p* = 0.029) (Table 1).

Top reasons for “why participate” in the campaign confirmed the themes gleaned from qualitative analysis of the original pledge survey. The top 4 reasons to participate represented more than 75% of responses and included (in this order) fairness or justice, being part of a movement or community, team diversity as an inherent good, and the sense that our institution should be a leader in gender equity.

In qualitative responses, people were pleased with the opportunity to build community and discuss equity concerns, sharing that “there were resources and peers to connect with to advance my and others' understanding of equality” (man, staff). Some were disappointed that they “didn't really do anything other than sign up” (woman, faculty), and others were pleased that they were able to “reflect on the current situation and adjust” (man, staff). “Half of the battle is awareness of one's beliefs and behavior and making changes a new habit. The other half is the larger social factors that reward self-interest and maintaining the status quo.” (gender nonconforming, faculty).

There were also opposing responses about the campaign's reach, with some impressed with the “reminder emails and general publicity about it” (woman, faculty), and alternatively “there was hype when pledges started, and I didn't hear or experience much effect since then” (woman, staff). Similarly, while some felt their leaders treated IWill as “just another ‘thing’ on their long list to do” (woman, staff), others observed that “leaders were early adopters” (woman, staff) and “high up leadership supported this effort” (woman, staff).

Overall, regarding the effectiveness of IWill, some were disappointed that they “didn't see or hear about any tangible outcomes” (woman, learner), or that the campaign did not change the culture enough: “I feel as if my specific pledge was helpful, and [that] gender discrimination and inequity are pervasive and demoralizing. …I think [IWill] is an interesting step that is not specifically effective in understanding or correcting the overall problem” (woman, faculty). Others were happy that the program was even begun, sharing that they were pleased “MCW is attempting to address and bring light to gender equity issues” (man, faculty). Additional quotes regarding things people were pleased and disappointed with are available in Table 4.

**Table 4.** Additional Quotes Under Things People Were Pleased or Disappointed with Regarding the Campaign

Things people were pleased with about the campaign		
Gender	Role	Quote
Leader engagement		
Woman	Faculty	MCW's leaders were visibly enthusiastic about participating and encouraging others to do participate, and for the right reasons (i.e., not just ‘checking a box’). I was also pleased at the broad and enthusiastic participation from many at MCW. Another thing is it spurred open and fascinating conversations about gender equity with colleagues that otherwise would not have happened.
Woman	Staff	I had the opportunities to make suggestions and talk with leaders who are committed to making gender equity a part of the MCW culture.
Reach and follow through		
Woman	Staff	It provided an opportunity for the MCW community to all get on the same page and together address gender equity in multiple ways.
Woman	Faculty	So many people were willing to look at gender equity, think about it, act in their own environment to improve it.
Man	Faculty	you are with honesty addressing this when it would be easy not to- values-based rather than driven by financial considerations
Woman	Faculty	There were a lot of reminder emails and general publicity about it.
Building community		
Woman	Faculty	so many other women I look up to struggled with similar things as me! It helped me feel like I'm not too far behind.
Woman	Staff	other team members come to me to discuss their concerns. I really like being able to see others grow in their positions and feel more of a part of the team.
Man	Staff	There were resources and peers to connect with to advance my and others understanding of equality
Institutional success		
Man	Staff	MCW is striving to be a leader in gender equity. It is right for the institution to do.
Woman	Staff	MCW even decided to run this campaign at all. Taking a stand on the issues and bringing awareness are the first steps toward change.
Woman	Faculty	It exists. It provides a short hand and permission for discussing expectations around equity of all kinds.
Changes in mental models		
Woman	Staff	I learned something about different gender identities and how important it is not to make assumptions but instead be open, aware, sensitive, and welcoming.
Man	Faculty	It made me consider more subtle examples of gender inequity.
Man	Staff	This was available to me so that I could be mindful of inequality at MCW. Otherwise, it is easy for me not to always be appropriately aware.

Things people were disappointed in regarding the campaign		
Gender	Role	Quote
Leader engagement		
Man	Faculty	More leaders were not intrinsically motivated to make pledges (rather than required/strongly encouraged to by [executive leadership])
Woman	Learner	I was disappointed that leadership did not comment on the project after maybe the intial pledge.
Woman	Faculty	Leaders pay lip service and when it comes to the nuts and bolts, they will not stand up for what is right.
Reach and follow through		
Woman	Faculty	I couldn't get a feel for how many pledges were honored. How is this information being tracked? What are the metrics of success?
Woman	Staff	More people didn't join, or there wasn't an open platform to talk about it with those I work directly with.
Man	Staff	In some regards it seems like the IWill program was put in place to check a box. Not saying that is the case, but many of these types of programs tend to be more of a, ‘Hey look at what we are doing’ vs. constructive change. I hope my perception is not true.
Competing commitments		
Woman	Faculty	I was often distracted from my pledge by other events. I wish I had spent more time looking at materials.
Woman	Staff	I was not able to attend any of the events that were held, which I feel would have been beneficial to me becoming more knowledgeable about gender equity in MCW
Woman	Faculty	There was not protected time to work on this-its another ‘thing’ that looks good but doesn't really have ‘teeth’ to be impactful.
Woman	Faculty	I just wasn't interested like i thought maybe i would be. i did not have much opportunity (or time and energy) to participate
Blocked by self or circumstances		
Woman	Staff	I didn't stay more committed to the process.
Man	Faculty	I did not do anything concrete

## Discussion

Our results demonstrate the impact of the IWill campaign and highlight barriers and facilitators to pledge-based campaigns at a large academic medical center, which could help guide others working to promote equity in academic medicine. To our knowledge, the IWill campaign is the first in the academic literature to describe this kind of pledge campaign and its follow-up, and it can serve as a benchmark for future similar campaigns.

### Pledge fulfillment by strategy

More than 80% of respondents both agreed they reflected on equity and fulfilled their pledge, indicating excellent engagement with those who pledged. In planning for the original campaign, the IWill team attempted to increase pledge fulfillment through several methods: public recognition of pledges and pledgemakers,^15,16^ providing information to assist pledge completion, and offering a variety of pledges to choose from. In this follow-up survey, we found that people who made public pledges did report fulfilling their pledge at significantly higher rates, but whether public pledges were a driver for completion, or there was an association between allowing your pledge to be public and thinking that you fulfilled it, remains unknown.

The IWill team provided written information to assist with pledge completion, but participants seemed to prefer more connection and group support—possibly linked to one of the top “whys” for participation: “I want to be part of a community that supports gender equity.” The IWill team hoped that offering a wide variety of pledges would increase fulfillment and engagement,^18^ but pledge type was not linked to fulfillment in either quantitative or qualitative responses. Having many pledges may have not only helped people find the right pledge for them but also may have been overwhelming or confusing.^21^ In addition, the large number of pledges made supporting a community for each pledge nearly impossible for the IWill team.

### Pledge fulfillment by group

Men were more likely to self-assess that they had fulfilled their pledge than women. This could be related to the increased tendency of men to make public pledges.^15,16^ Alternatively, like others with privilege, men may be less likely to recognize gender inequities, or to actively reframe inequities they do acknowledge.^22,23^ Alternatively, men may be more optimistic about prospects for gender equality,^24^ or feel more in control of their surroundings, an idea supported by the preponderance of men in this study that felt they had the power to create gender equity.

Women endorsed fulfilling their pledges at lower rates than men or learners. Increased saliency of systems level or cultural barriers may promote feeling less successful and less empowered to make change. Examples shared in qualitative responses included a faculty-staff power differential (most staff at our institution are women) and the negative perception of women who act like “feminists.” Women may also be less likely to promote their successes,^25,26^ or to feel confident in their achievements.^27^

Furthermore, many women shared in qualitative responses that their quest for gender equity was a lifelong endeavor—indicating that, for women, this is a topic that will continue to bring challenges. Finally, while it was not expressed in qualitative comments, it is possible that women feel overburdened by other areas of their lives. For example, they may be doing other equity work^28^ or citizenship tasks^29^ at work, or doing more work at home,^6^ leaving little time or energy remaining for pledge fulfillment.

Like the men in our study, learners were more optimistic about both pledge fulfillment and change-making ability. They may be experiencing a more favorable environment, have not had the opportunity to become jaded by life experiences, or are generally more optimistic about their ability to create change.^30^

### Empowerment and culture

The campaign was based on Kania's systems of culture change model, and focused on supporting culture change at the semi-implicit and implicit levels.^12,13^ The IWill team found that most pledge makers fulfilled their pledges by striving to flatten power dynamics and altering their mental models of equity. Despite the campaign's efforts, culture and hierarchy represented a striking barrier, represented by leader behaviors and choices, entrenchment of the staff-faculty hierarchy, and perceptions of men and women regarding workplace norms—likely linked to the finding that only 55% of men and 40% of women felt the IWill campaign improved gender equity. While disappointing, it was additionally clear that the polarity of conditions in pledge makers' immediate environments—culture at the departmental or unit level and leader engagement and support—directly affected respondents' views of their own success and ability to effect change.

### Limitations

The IWill campaign reached 1405 (16%) of all MCW learners, staff, and faculty, and therefore our results are may not represent our full population, or other medical institutions. We did not survey those who did not participate, and therefore do not have direct access to why people chose not to participate. Based on anecdotal comments, the IWill team believes that competing interests (such as clinical work) and a belief that gender equity is no longer a salient issue contributed to the lack of responses. In addition, while we hoped to have a positive impact on gender equity, it was not the expectation that a onetime campaign would have profound impacts, but rather be one step in addressing change within the institution. Despite these limitations, the lessons learned from IWill can help inform future work both at our institution and for others.

### Future directions

The IWill team sees three critical actions to improve future campaigns: providing key administrative supports, increasing engagement with culture, and connecting to the “why.”

For any campaign to be successful, it must reach and engage people. While we consider our initial engagement to be a great first step, future iterations will need to garner more pledge makers and engage those people in pledge fulfillment. As key administrative supports, the IWill team plans to continue public pledges, but provide fewer written materials, streamline the pledge process, and decrease the number of pledges. The team believes that redirecting efforts toward a year-round format will better support pledge makers, increasing both engagement and fulfillment.

Improving culture is critical to success and targeting departments' specific needs seems both strategic, given limited resources, and important, given the varied cultures pledge makers were experiencing. With this in mind, the IWill team plans to expand to departmental and institution-wide initiatives, focusing on semiexplicit (power dynamics) and explicit (policy and funds flow) changes.^13^ Expanding IWill to departments and schools, in addition to being key elements of culture change, could also garner additional pledge makers (especially our optimistic learner group) as people see real change come directly to their areas.

Key learnings regarding critical areas of culture include the faculty-staff hierarchy and beliefs in pledge success as a marker for culture change. Hierarchy is often discussed within and between faculty and learners,^31^ but the faculty-staff divide is both understudied and pervasive. The IWill team will direct its efforts toward supporting staff (the majority of whom are women) through specific programming intended to understand and empower their voices.

The IWill team was surprised by the relationship between gender and endorsement of pledge fulfillment. While women's confidence in their own successes could play a role in their perceptions of pledge fulfillment, the IWill team agrees that altering mental models and creating equitable environments have no quick fix^32^ and exploring ways to encourage complex processes rather than “success” is a continual goal.^33^ Because these finding were linked to qualitative data, future investigations should seek to uncover whether there are systematic gender differences in the perceptions of barriers.

Responses to the “why participate” question suggested that future campaigns could be more effective if participants felt as if they were part of a movement that seeks to promote fairness and fosters institutional pride. The IWill team plans to provide administrative support for collaborative efforts, focus future pledges on promoting justice and diverse teams, and emphasize institutional pride in its communications, building a culture in which all genders thrive.

## Supplementary Material

Supplemental data

## Supplementary Material

Supplementary Appendix SA

## Abbreviations Used

ACA: Affordable Care Act
ANOVA: analysis of variance
AWSM: Advancement of Women in Science and Medicine
COVID-19: coronavirus disease 2019
CQA: Consensual Qualitative Analysis
MCW: Medical College of Wisconsin's

## References

[1] 2021 FACTS: Applicants and Matriculants Data. AAMC. Available from: https://www.aamc.org/data-reports/students-residents/interactive-data/2021-facts-applicants-and-matriculants-data [Last accessed: October 7, 2022].

[2] U.S. Medical School Faculty Trends: Promotion and Attrition. AAMC. Available from: https://www.aamc.org/data-reports/faculty-institutions/interactive-data/https/wwwaamcorg/data-reports/interactive-data/us-medical-school-faculty-trends-promotion-attrition [Last accessed: October 7, 2022].

[3] Odei BC, Seldon C, Fernandez M, et al. Representation of women in the leadership structure of the US health care system. JAMA Netw Open 2021;4(11):e2136358; doi: 10.1001/jamanetworkopen.2021.36358 34842928 PMC8630566

[4] Travis EL, Doty L, Helitzer DL. Sponsorship: A path to the academic medicine C-suite for women faculty? Acad Med 2013;88(10):1414–1417; doi: 10.1097/ACM.0b013e3182a35456 23969365

[5] Periyakoil VS, Chaudron L, Hill EV, et al. Common types of gender-based microaggressions in medicine. Acad Med 2020;95(3):450–457; doi: 10.1097/ACM.0000000000003057 31688038

[6] Jolly S, Griffith KA, DeCastro R, et al. Gender differences in time spent on parenting and domestic responsibilities by high-achieving young physician-researchers. Ann Intern Med 2014;160(5):344–353; doi: 10.7326/M13-0974 24737273 PMC4131769

[7] Ellinas EH, Ark TK, Kaljo K, et al. Winners and losers in academic productivity during the COVID-19 pandemic: Is the gender gap widening for faculty? J Womens Health 2002 2022;31(4):487–494; doi: 10.1089/jwh.2021.0321 34935469

[8] Ackerman-Barger K, Jacobs NN, Orozco R, et al. Addressing microaggressions in academic health: A workshop for inclusive excellence. MedEdPORTAL 2021;17:11103; doi: 10.15766/mep_2374-8265.11103 33598543 PMC7880252

[9] Carnes M, Devine PG, Baier Manwell L, et al. The effect of an intervention to break the gender bias habit for faculty at one institution: A cluster randomized, controlled trial. Acad Med 2015;90(2):221–230; doi: 10.1097/ACM.0000000000000552 25374039 PMC4310758

[10] Silver J. She Leads Healthcare. She Leads Healthcare. Available from: https://sheleadshealthcare.com [Last accessed: October 7, 2022].

[11] Umphress E, Thomas J. Applying Procedural Justice to Sexual Harassment Policies Processes and Practices. 2022. Available from: https://www.nationalacademies.org/news/2022/04/applying-procedural-justice-to-sexual-harassment-policies-processes-and-practices [Last accessed: October 7, 2022].

[12] Maurana CA, Raymond JR, Kerschner JE, et al. The IWill MCW campaign: Individual actions to advance gender equity. Acad Med 2021;96(6):817–821; doi: 10.1097/ACM.0000000000004016 33637663

[13] Kania J, Kramer M, Senge P. The Water of Systems Change. Seattle, WA: FSG; 2018. Available from: https://www. fsg. org/publications [Last accessed: April 10, 2023].

[14] Dearing JW, Cox JG. Diffusion of innovations theory, principles, and practice. Health Aff (Millwood) 2018;37(2):183–190; doi: 10.1377/hlthaff.2017.1104 29401011

[15] Cotterill S, John P, Richardson L. The impact of a pledge request and the promise of publicity: A randomized controlled trial of charitable donations. Soc Sci Q 2013;94(1):200–216; doi: 10.1111/j.1540-6237.2012.00896.x

[16] Sutan A, Grolleau G, Mateu G, et al. “Facta non verba”: An experiment on pledging and giving. J Econ Psychol 2018;65:1–15; doi: 10.1016/j.joep.2018.01.006

[17] Gollwitzer PM, Sheeran P. Implementation intentions and goal achievement: A meta-analysis of effects and processes. Adv Exp Soc Psy 2006;38:69–119; doi: 10.1016/S0065-2601(06)38002-1

[18] Brower T. The Power of Choice and What Matters Most for the Future of Work. Forbes. 2021. Available from: https://www.forbes.com/sites/tracybrower/2021/02/21/the-power-of-choice-and-what-matters-most-for-the-future-of-work [Last accessed: October 2, 2022].

[19] Harris PA, Taylor R, Minor BL, et al. The REDCap consortium: Building an international community of software platform partners. J Biomed Inform 2019;95:103208; doi: 10.1016/j.jbi.2019.103208 31078660 PMC7254481

[20] Hill CE, Knox S, Thompson BJ, et al. Consensual qualitative research: An update. J Couns Psychol 2005;52(2):196–205; doi: 10.1037/0022-0167.52.2.196

[21] Schwartz B. The Paradox of Choice: Why More Is Less, Revised Edition. Revised edition. Ecco: New York; 2016.

[22] Schwiter K, Nentwich J, Keller M. Male privilege revisited: How men in female-dominated occupations notice and actively reframe privilege. Gend Work Organ 2021;28(6):2199–2215; doi: 10.1111/gwao.12731

[23] Peretz T. Seeing the invisible knapsack: Feminist men's strategic responses to the continuation of male privilege in feminist spaces. Men Masculinities 2020;23(3–4):447–475; doi: 10.1177/1097184X18784990

[24] Horowitz JM, Fetterolf J. Worldwide Optimism About Future of Gender Equality, Even as Many See Advantages for Men. Pew Research Center's Global Attitudes Project. 2020. Available from: https://www.pewresearch.org/global/2020/04/30/worldwide-optimism-about-future-of-gender-equality-even-as-many-see-advantages-for-men [Last accessed: September 30, 2022].

[25] Ellinas EH, Kaljo K, Patitucci TN, et al. No room to “Lean In”: A qualitative study on gendered barriers to promotion and leadership. J Womens Health 2019;28(3):393–402; doi: 10.1089/jwh.2018.7252 30481114

[26] Exley CL, Kessler JB. The gender gap in self-promotion (No. w26345). National Bureau of Economic Research 2019. Available from: http://www.nber.org/papers/w26345 [Last accessed: April 10, 2023].

[27] Shipman KK Claire. The Confidence Gap. The Atlantic. 2014. Available from: https://www.theatlantic.com/magazine/archive/2014/05/the-confidence-gap/359815 [Last accessed: October 11, 2022].

[28] Santhosh L, Keenan BP, Jain S. The “Third Shift”: A path forward to recognizing and funding gender equity efforts. J Womens Health 2020;29(11):1359–1360; doi: 10.1089/jwh.2020.8679 PMC8020533 32744885

[29] Armijo PR, Silver JK, Larson AR, et al. Citizenship tasks and women physicians: Additional woman tax in academic medicine? J Womens Health 2002 2021;30(7):935–943; doi: 10.1089/jwh.2020.8482 33202161

[30] Generation Z: Building a Better Normal. Wunderman Thompson. 2020. Available from: https://www.wundermanthompson.com/insight/generation-z [Last accessed: October 2, 2022].

[31] Vanstone M, Grierson L. Thinking about social power and hierarchy in medical education. Med Educ 2022;56(1):91–97; doi: 10.1111/medu.14659 34491582

[32] Dugan J. Beware of Equity Traps and Tropes. ASCD. 2021. Available from: https://www.ascd.org/el/articles/beware-of-equity-traps-and-tropes [Last accessed: November 8, 2022.].

[33] Bates C, Gordon L, Travis E, et al. Striving for gender equity in academic medicine careers: A call to action. Acad Med J Assoc Am Med Coll 2016;91(8):1050–1052; doi: 10.1097/ACM.0000000000001283 PMC5954825 27332868

