# Supplementary material for: Lessons and Future Directions for a Gender Equity Pledge Campaign
Source: Womens Health Rep (New Rochelle). 2023 May 29;4(1):251–61. doi: 10.1089/whr.2022.0111 (PMC10240325; doi:10.1089/whr.2022.0111)
Supplement: Supplemental data [file Supp_DataS1.pdf]

Clinical & Translational Science Institute
of Southeast Wisconsin

Medical College of Wisconsin
Clinical and Translational Science Institute

MCW Gender Equity Campaign PID 1276

Codebook

Data Dictionary Codebook

07/31/2020 3:29pm

Collapse all instruments

	#	Variable / Field Name	Field Label<br>*Field Note*	Field Attributes (Field Type, Validation, Choices, Calculations, etc.)
Instrument: **Survey** (survey) Enabled as survey				Expand
Instrument: **Buffer** (buffer) Enabled as survey				Expand
Instrument: **Buffer2** (buffer_2) Enabled as survey				Expand
Instrument: **Followup** (test) Enabled as survey				Collapse
	43	section1	PART 1. INTRODUCTION Thank you for demonstrating your commitment to gender equity through the IWillMCW campaign! Throughout this document we refer to the IWill pledge you made last fall. The IWill Team's original plan was to release this follow-up survey in March 2020. Although COVID-19 delayed the survey's release, the pandemic has heightened our awareness that gender inequity exists and must be addressed. We greatly value your experience with IWill, whether you view your experience as entirely successful, or entirely not. We would appreciate your feedback regarding the pledges themselves, the support material, and anything else we should start or stop doing to support your success. Please Note: Throughout this survey we define gender equity as "fairness of treatment for all genders, according to their respective needs." In these sense, gender equity encompasses all pledge categories, from parental leave through micro-aggressions or workplace ally.	descriptive
	44	section2	Section Header:<br>PART 2. IRB FOLLOW UP To begin, we would like your input on sharing your IWill responses -- both to our original campaign last fall, and to future work. As you know, your IWill pledge was not anonymous. You had the choice to share your pledge either just with the IWill team, or more broadly within MCW (your choice). The IWill team has analyzed what you shared, and presented the results collectively; for example, how many pledges were made, or themes from the answers to write-in questions. Some of the ideas shared in IWill were extremely powerful, and we would like to be able to share that information using direct anonymized quotes without using your name.	descriptive
	45	share_open	Please check the box that reflects your opinion regarding anonymous quote sharing:	radio, Required<br>1 Yes, You may share a direct quote of my IWill responses without using my name<br>2 No, Please do not quote me directly, even without my name

	46	share_open_other	Please share any additional comments your might have on the above	notes
	47	section3	Section Header:<br>PART 3. RESOURCES USED Thank you again for making an IWill pledge! In this section, please refer to the pledge you made in Fall 2019.	descriptive
	48	i	I remember my IWill pledge.	radio<br>1 Strongly Agree<br>2 Agree<br>3 Neutral<br>4 Disagree<br>5 Strongly Disagree
	49	automated_email	After your pledge, did you receive an automated email that contained your pledge and some information (bullet points) regarding the pledge?	radio<br>1 Yes<br>2 No<br>3 I don't remember
	50	click_link	If Yes, Did you click on the link to more materials?	yesno<br>1 Yes<br>0 No
	51	useful	If Yes, Were those materials in the "Box" useful?	yesno<br>1 Yes<br>0 No
	52	useful_other	Please comment on the box materials	notes
	53	section4	Section Header:<br>PART 4. FULFILLING YOUR PLEDGE Please share your thoughts on the following statements Please Note: Throughout this survey we define gender equity as "fairness of treatment for all genders, according to their respective needs." In this sense, gender equity encompasses all pledge categories, from parental leave through micro-aggressions or workplace ally.	descriptive
	54	learned_something	Through the IWill process, I learned something new about gender equity	radio<br>1 Strongly Agree<br>2 Agree<br>3 Neutral<br>4 Disagree<br>5 Strongly Disagree
	55	reflected	Through the IWill process, I was able to take some time to reflect about gender equity in my environment	radio<br>1 Strongly Agree<br>2 Agree<br>3 Neutral<br>4 Disagree<br>5 Strongly Disagree
	56	acted	Through the IWill process, I acted to promote gender equity	radio<br>1 Strongly Agree<br>2 Agree<br>3 Neutral<br>4 Disagree<br>5 Strongly Disagree

	57	became_aware	I have become aware of a gender inequity that I did not see before	radio<br>1 Strongly Agree<br>2 Agree<br>3 Neutral<br>4 Disagree<br>5 Strongly Disagree
	58	not_original_pledge	I worked on or acted on a gender-equity activity that was not my original IWill pledge	radio<br>1 Strongly Agree: I worked on an alternative pledge<br>2 Agree: I worked on a pledge that was related but not exactly my pledge<br>3 Neutral<br>4 Disagree: I don't think I worked on an alternative pledge<br>5 Strongly Disagree: I did not work on an alternative pledge
	59	fulfilled	I was able to fulfill my IWill Pledge	radio<br>1 Definitely yes<br>2 Maybe<br>3 Neutral<br>4 Maybe not<br>5 Definitely not
	60	fulfilled_other	Please tell us why you feel you did or did not fulfill your pledge. Specific examples appreciated.	notes
	61	section5	Section Header:<br>PART 5. BARRIERS and SUPPORTS	descriptive
	62	barriers	Please describe any barriers to completing your pledge (click all that apply)	checkbox<br>1 barriers___1 Could not access learning materials<br>2 barriers___2 Learning materials were not helpful<br>3 barriers___3 No or insufficient activities to attend<br>4 barriers___4 No opportunities to act on my pledge<br>5 barriers___5 I forgot about the pledge<br>6 barriers___6 Someone in my environment prevented me from completing my pledge<br>7 barriers___7 Other
	63	barriers_other	Please comment on barriers to fulfilling your pledge -- are we missing any?	notes
	64	support	Please describe any supports to completing your pledge (click all that apply)	checkbox<br>1 support___1 Learning materials provided by IWill<br>2 support___2 Learning materials from elsewhere<br>3 support___3 An activity I attended<br>4 support___4 A person who knew I had pledged and helped me fulfill it<br>5 support___5 Other
	65	support_other	Please comment on supports to fulfilling your pledge -- are we missing any?	notes

	66	section7	Section Header:<br>PART 6. ENGAGING OTHERS IN EQUITY	descriptive
	67	communicate	Did you communicate with anyone about IWill? (click all that apply)	checkbox<br>1 communicate___1 My family members<br>2 communicate___2 Others I work with<br>3 communicate___3 Other
	68	communicate_other	Other (please specify)	notes
	69	encourage	Did you encourage anyone to make a pledge?	yesno<br>1 Yes<br>0 No
	70	encourage_other	Please tell us about that experience	notes
	71	section6	Section Header:<br>PART 7. GAINING UNDERSTANDING	descriptive
	72	why_intro	We received many wonderful responses to why participants wanted to join the IWill campaign and want to increase our understanding on this topic. If you would, please check the Most Important reason(s) from the list below. You may select up to THREE. If we missed your reason, please add it at the bottom!	descriptive
	73	top_why1	It's fair, just or right	radio (Matrix - ranking)<br>1 Top 1: 1st Reason<br>2 Top 2: 2nd Reason<br>3 Top 3: 3rd Reason
	74	top_why2	Diverse teams work better	radio (Matrix - ranking)<br>1 Top 1: 1st Reason<br>2 Top 2: 2nd Reason<br>3 Top 3: 3rd Reason
	75	top_why3	IWill MCW is a good approach	radio (Matrix - ranking)<br>1 Top 1: 1st Reason<br>2 Top 2: 2nd Reason<br>3 Top 3: 3rd Reason
	76	top_why4	MCW should be a leader in gender equity	radio (Matrix - ranking)<br>1 Top 1: 1st Reason<br>2 Top 2: 2nd Reason<br>3 Top 3: 3rd Reason
	77	top_why5	A peer or leader encouraged me to pledge	radio (Matrix - ranking)<br>1 Top 1: 1st Reason<br>2 Top 2: 2nd Reason<br>3 Top 3: 3rd Reason
	78	top_why6	I have personally experienced inequity or harassment	radio (Matrix - ranking)<br>1 Top 1: 1st Reason<br>2 Top 2: 2nd Reason<br>3 Top 3: 3rd Reason
	79	top_why7	Colleagues have experienced inequity or harassment	radio (Matrix - ranking)<br>1 Top 1: 1st Reason<br>2 Top 2: 2nd Reason<br>3 Top 3: 3rd Reason

	80	top_why9	I have a daughter (or family member) and would like to see them treated fairly	radio (Matrix - ranking)<br>1 Top 1: 1st Reason<br>2 Top 2: 2nd Reason<br>3 Top 3: 3rd Reason
	81	top_why10	I believe in supporting parents	radio (Matrix - ranking)<br>1 Top 1: 1st Reason<br>2 Top 2: 2nd Reason<br>3 Top 3: 3rd Reason
	82	top_why11	I want to learn (e.g., about microaggressions or gender equity)	radio (Matrix - ranking)<br>1 Top 1: 1st Reason<br>2 Top 2: 2nd Reason<br>3 Top 3: 3rd Reason
	83	top_why12	I want to be part of a community or movement that supports gender equity	radio (Matrix - ranking)<br>1 Top 1: 1st Reason<br>2 Top 2: 2nd Reason<br>3 Top 3: 3rd Reason
	84	other	Other	radio (Matrix - ranking)<br>1 Top 1: 1st Reason<br>2 Top 2: 2nd Reason<br>3 Top 3: 3rd Reason
	85	why_other_top	Other (please specify)	notes
	86	why_intro2	Were any of the listed reason NOT an important factor for why you pledged or joined the IWill campaign? (Select all the apply)	checkbox<br>1 why_intro2___1 It's fair, just or right<br>2 why_intro2___2 Diverse teams work better<br>3 why_intro2___3 IWill MCW is a good approach<br>4 why_intro2___4 MCW should be a leader in gender equity<br>5 why_intro2___5 A peer or leader encouraged me to pledge<br>6 why_intro2___6 I have personally experienced inequity or harassment<br>7 why_intro2___7 Colleagues have experienced inequity or harassment<br>8 why_intro2___8 I have a daughter (or family member) and would like to see them treated fairly<br>9 why_intro2___9 I believe in supporting parents<br>10 why_intro2___10 I want to learn (e.g., about microaggressions or gender equity)<br>11 why_intro2___11 I want to be part of a community or movement that supports gender equity
	87	power	I have the power to create gender equity in my work environment	radio<br>1 Strongly Agree<br>2 Agree<br>3 Neutral<br>4 Disagree<br>5 Strongly Disagree

	88	iwill_process_other	Please tell us why you feel (or don't feel) that you have the power to affect gender equity in your area. One example appreciated.	notes
	89	overall_comments_please_sh	Section Header:<br>PART 8: OVERALL COMMENTS:Please share your thoughts on IWill MCW.	descriptive
	90	hoped	I was able to gain what I hoped I would from IWill MCW	radio<br>1 Strongly Agree<br>2 Agree<br>3 Neutral<br>4 Disagree<br>5 Strongly Disagree
	91	improved	I believe that IWill MCW has improved gender equity at MCW	radio<br>1 Strongly Agree<br>2 Agree<br>3 Neutral<br>4 Disagree<br>5 Strongly Disagree
	92	covid_pleasenote	Please Note: Throughout this survey we define gender equity as "fairness of treatment for all genders, according to their respective needs." In this sense, gender equity encompasses all pledge categories, from parental leave through micro-aggressions or workplace ally.	descriptive
	93	pleased_other	Regarding my IWill MCW experience, I was pleased that:	notes
	94	disappointed_other	Regarding my IWill MCW experience, I was disappointed that:	notes
	95	covid1	Since the onset of the COVID-19 crisis, working with my IWill pledge has been	radio<br>1 Much easier than before COVID<br>2 Easier than before COVID<br>3 The same<br>4 Harder than before COVID<br>5 Much harder than before COVID
	96	covid2	Since the onset of the COVID-19 crisis, promoting gender equity has been	radio<br>1 Much easier than before COVID<br>2 Easier than before COVID<br>3 The same<br>4 Harder than before COVID<br>5 Much harder than before COVID
	97	covid3	Since the onset of the COVID-19 crisis, please describe your experience of gender equity in your professional work life. With the pandemic, my my professional work feels:	radio<br>1 Much more equitable<br>2 More equitable<br>3 The same<br>4 Less equitable<br>5 Much less equitable
	98	covid_open	If you would like to, please share an experience or thoughts regarding gender equity during the pandemic. We would be especially appreciative of things that you think are important to share with colleagues or leadership.	notes
	99	section8	Section Header:<br>PART 9. FUTURE Please share your thoughts about the future of IWill MCW	descriptive

	100	another_opportunity	I would like for there to be another opportunity to make a gender equity pledge	radio<br>1 Strongly Agree<br>2 Agree<br>3 Neutral<br>4 Disagree<br>5 Strongly Disagree
	101	same_pledges	If there is another opportunity to pledge, the pledges should be the same as the previous set	radio<br>1 Strongly Agree<br>2 Agree<br>3 Neutral<br>4 Disagree<br>5 Strongly Disagree
	102	future_thoughts	Please share any thoughts about future directions for IWill.	notes
	103	thankyou	Thank you for completing this IWill MCW follow up! We appreciate your feedback, and hope to continue to partner with you in the future as we work toward gender equity.	descriptive
	104	fu_comp_date	Followup Completion Date	text<br>Field Annotation: @HIDDEN-SURVEY @TODAY
	105	test_complete	Section Header: *Form Status*<br>Complete?	dropdown<br>0 Incomplete<br>1 Unverified<br>2 Complete

Instrument: **SurveyMessage** (surveymessage) Enabled as survey — Expand

Instrument: **workplace1** (workplace1) Enabled as survey — Expand

Instrument: **workplace2** (workplace2) Enabled as survey — Expand

Instrument: **workplace3** (workplace3) Enabled as survey — Expand

Instrument: **workplace4** (workplace4) Enabled as survey — Expand

Instrument: **workplace5** (workplace5) Enabled as survey — Expand

Instrument: **parentsupport1** (parentsupport1) Enabled as survey — Expand

Instrument: **parentsupport2** (parentsupport2) Enabled as survey — Expand

Instrument: **parentsupport3** (parentsupport3) Enabled as survey — Expand

Instrument: **parentsupport4** (parentsupport4) Enabled as survey — Expand

Instrument: **microaggressions1** (microaggressions1) Enabled as survey — Expand

Instrument: **microaggressions2** (microaggressions2) Enabled as survey — Expand

Instrument: **microaggressions3** (microaggressions3) Enabled as survey — Expand

Instrument: **belonging1** (belonging1) Enabled as survey — Expand

Instrument: **belonging2** (belonging2) Enabled as survey — Expand

Instrument: **belonging3** (belonging3) Enabled as survey — Expand

Instrument: **belonging4** (belonging4) Enabled as survey — Expand

Instrument: **harassment1** (harassment1) Enabled as survey — Expand

Instrument: **harassment2** (harassment2) Enabled as survey — Expand

Instrument: **otherpledge** (otherpledge) Enabled as survey

Expand
